# Synergistic and individual effects of RNase E, II, and R in the regulation of *Escherichia coli* growth and metabolism

**DOI:** 10.1128/aem.00090-26

**Published:** 2026-06-26

**Authors:** Marvin Ramos-Hue, Marjorie Audonnet, Maïssanne Dahmani, Mathilde Burck, Muriel Cocaign-Bousquet, Laurence Girbal

**Affiliations:** 1 TBI, Université de Toulouse, CNRS, INRAE, INSA, Toulouse, France; Washington University in St Louis, St. Louis, Missouri, USA

**Keywords:** RNases, metabolism, *E. coli *BL21(DE3), growth rate, glycogen, acetate overflow

## Abstract

Messenger RNA levels, crucial for cell survival and adaptation, are regulated through their degradation by ribonucleases (RNases). Although the molecular mechanisms of RNases in *Escherichia coli* are established, the broader effects of RNases on growth and metabolism remain unclear. Here, the roles of three RNases, E, II, and R, were examined individually and in combination in double and triple mutants. Growth behaviors and metabolic changes were analyzed on different carbon sources and under recombinant protein production conditions. C-terminal truncation of RNase E was unexpectedly found to have strong effects, promoting carbon-storage metabolism, thereby leading to glycogen accumulation, especially with glucose as a carbon source. Even more surprisingly, it accelerated growth on xylose. Synergistic interactions between the three RNases were also identified. Deleting RNase R amplified glycogen accumulation in the RNase E mutant, and further increased its growth rate. All three RNases were found to significantly contribute to acetate overflow regulation, with synergy between RNase E and R. Combined mutations had additional benefits under protein production conditions. Compared with the parental strain, the double mutant with both RNase E truncation and RNase II deletion produced up to twice as much recombinant protein, grew faster on xylose, and produced more glycogen on glucose. Overall, this work shows that RNase E, II, and R act both independently and synergistically in controlling *E. coli* growth and metabolism across carbon sources and bioproduction conditions. These findings highlight the strong relationship between RNA degradation and cell physiology and offer perspectives for engineering optimized microbial chassis in biotechnology.

**IMPORTANCE:**

Messenger RNA degradation is a fundamental layer of gene regulation; however, its system-level impact on bacterial physiology remains poorly understood. Here, we show that RNase E, RNase II, and RNase R act both independently and synergistically to control *Escherichia coli* growth and metabolism across diverse carbon sources and bioproduction conditions. Unexpectedly, C-terminal truncation of RNase E, either alone or in combination with RNase II or RNase R, modulates glycogen and acetate metabolism and improves growth performance. Enhanced recombinant protein production was also observed, and the underlying mechanism was investigated. By directly linking the RNA degradation machinery to metabolic regulation and cellular performance, this work highlights RNases as powerful and underexploited targets for engineering improved microbial chassis for biotechnology.

## INTRODUCTION

*Escherichia coli*’s complex degradation machinery, involving endoribonucleases (RNase E, G, P, III, and Z) and exoribonucleases (RNase II and R, polynucleotide phosphorylase [PNPase], and oligoribonuclease) (1), allows it to adjust mRNA levels in response to environmental changes. Despite detailed knowledge of RNase mechanisms and their sequential activities in mRNA degradation (1–4), the individual and collective effects of these enzymes on *E. coli* cell growth and metabolism remain unclear.

The best-characterized RNases at the molecular level include the key endoribonuclease RNase E and the major exoribonucleases, RNase II, PNPase, and RNase R. RNase E initiates most mRNA degradation in *E. coli* via endoribonucleolytic cleavage. RNase E is membrane-anchored and forms the RNA degradosome by associating with PNPase, RNA helicase B, and the glycolytic enzyme enolase through its C-terminal scaffold region. Truncation of the C-terminal region of RNase E results in the formation of an enzyme that is unable to assemble the RNA degradosome but remains catalytically active. RNase II and RNase R are 3′-exoribonucleases that, in addition to the degradosome-associated PNPase, contribute to the conversion of RNase E cleavage products into oligonucleotides. RNase II and RNase R both belong to the RNase II/RNB family of hydrolytic exoribonucleases. RNase II exclusively degrades single-stranded RNA, whereas RNase R can also degrade structured RNA, provided there is a 3′-end overhang.

Surprisingly perhaps, growth rate regulation has never been directly studied in single and combined RNase mutants. All that is known in this context is the viability (all-or-nothing growth response) of single and double RNase mutants: RNase E is known to be essential for *E. coli* cell survival, although mutants that lack the C-terminal region remain viable (5). Deletion of RNase II or RNase R does not compromise cell viability, nor does the double deletion of RNase II and RNase R; however, double RNase R plus PNPase and RNase II plus PNPase mutants are not viable (6–8).

Information is also lacking on how RNase mutations, alone or in combination, affect the overall metabolic landscape. RNases seem to influence metabolic regulation by modulating specific pathways and stress responses (9). In particular, RNase E regulates the stability of certain mRNAs involved in sugar uptake, acetate metabolism, and response to iron starvation via interaction with the small RNAs SgrS, SdhX, and RyhB, respectively (10–14). RNase II and RNase R contribute to the growth of *E. coli* at low temperatures: RNase II can compensate for the essential role of PNPase (15), but not of RNase R (16), while RNase R contributes to cold-temperature acclimation as part of a two-member mRNA surveillance system (17).

The nature and scale of the effects of RNases on *E. coli* growth and metabolism are therefore unclear. The aim of this study was to elucidate the individual and combined roles of three RNases: the endoribonuclease RNase E, and two exoribonucleases, RNase II and RNase R. We constructed seven different mutant strains: three single mutants with either C-terminal truncated RNase E, RNase II deletion, or RNase R deletion, the three corresponding double mutants, and the triple mutant. We analyzed growth and metabolic dynamics of the strains on two carbon sources, glucose (preferred) and xylose (non-preferred), and under recombinant protein production conditions. Our results reveal the previously unrecognized, robust, and synergistic roles of these RNases in shaping *E. coli* cellular physiology.

## RESULTS

### RNase E, II, and R contribute to the regulation of acetate overflow with limited effects on growth and carbon utilization on glucose

**Fig 1:**
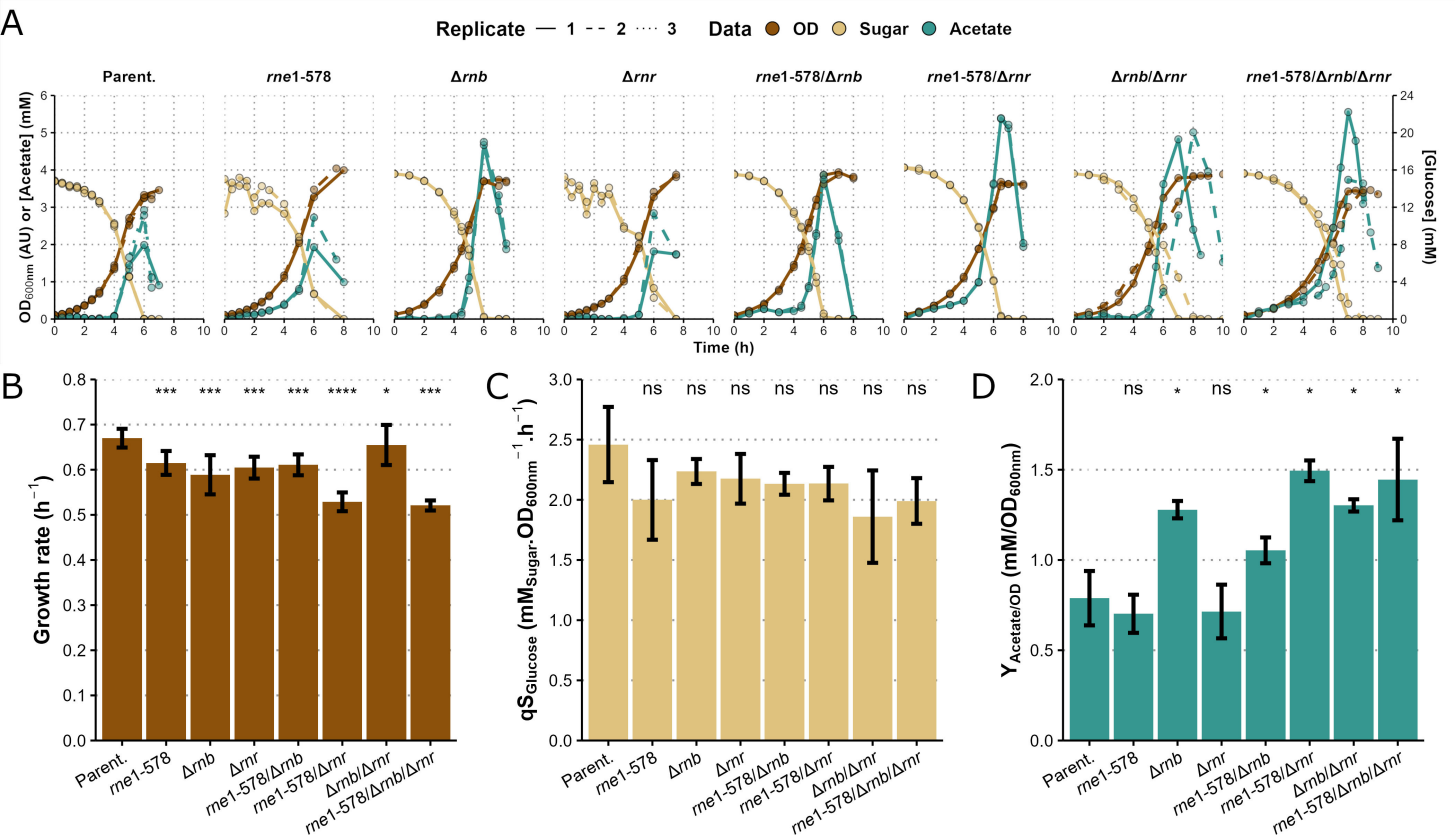
Kinetic analyses of *E. coli* RNase E, II, and R mutants grown on glucose in flasks. (**A**) Optical density (OD; brown), glucose concentration (beige), and acetate concentration (green) as a function of time (*n* ≥ 2 biological replicates). (**B**) Maximum growth rate (*n* ≥ 4 biological replicates) and (**C**) maximum specific glucose uptake rate (*n* = 2 biological replicates × 3 exponential-phase points) determined in the exponential growth phase. (**D**) Maximum acetate yield (*n* = 2 biological × 2 technical replicates) determined at the acetate peak. Bars represent mean ± SD.

To investigate how the studied RNases regulate *E. coli* growth and metabolism on glucose, we measured growth rates, carbon source consumption, and acetate production in all RNase mutants relative to the *E. coli* BL21(DE3) parental strain: the three single mutants with truncation of the C-terminal domain of RNase E (*rne*1-578), with deletion of RNase II (Δ*rnb*), or with deletion of RNase R (Δ*rnr*), the three corresponding double mutants, and the triple mutant. The seven mutant strains, along with the parental strain, were grown on glucose. Kinetic analyses were performed based on macroscopic data (Fig. 1A) and kinetic parameter estimates (Fig. 1B through D).

Maximal growth rates (µ) on glucose were only slightly reduced in the mutants compared with the parental strain (Fig. 1B). The reduction of growth rate was approximately 10% for the three single mutants and two double mutants, *rne*1-578/Δ*rnb* and Δ*rnb*/Δ*rnr* (average µ, 0.61 ± 0.02 h^−1^, compared with 0.67 ± 0.02 h^−1^ for the parental strain) and about 20% for the *rne*1-578/Δ*rnr* double mutant and for the triple mutant (average µ of 0.53 ± 0.01 h^−1^). Changes in the maximal glucose consumption rate (qS_glucose_) in the mutants were also small and generally mirrored those in the growth rate (Fig. 1C). In contrast, a strong increase of 33%–89% in acetate production capacity was measured in the Δ*rnb* single mutant, all double mutants, and the triple mutant (Fig. 1D). Acetate accumulation was highest in the *rne*1-578/Δ*rnr* mutant (peak concentration, 5.38 ± 0.01 mM, compared with 2.57 ± 0.51 mM for the parental strain; Fig. 1A), as was acetate yield (89% higher than for the parental strain; Fig. 1D). The increase in acetate accumulation in *rne*1-578/Δ*rnr* was unexpected, since both single mutants showed no change in acetate production compared with the parental strain, a result that clearly points to a synergistic interaction between RNase E and R that promotes acetate production. The increase in acetate production in the *rne*1-578/Δ*rnr* mutant was associated with a decrease in growth rate compared with the parental strain. This is contrary to the usual positive correlation between acetate production and µ when acetate overflow occurs at high growth rates (18). Altogether, these results indicate that RNase E, II, and R have limited effects on growth and carbon utilization on glucose but strongly regulate acetate overflow metabolism, individually for RNase II and synergistically for RNase E and R.

### C-terminal truncation of RNase E accelerates growth and sugar uptake on xylose, alone and synergistically with RNase R deletion

**Fig 2:**
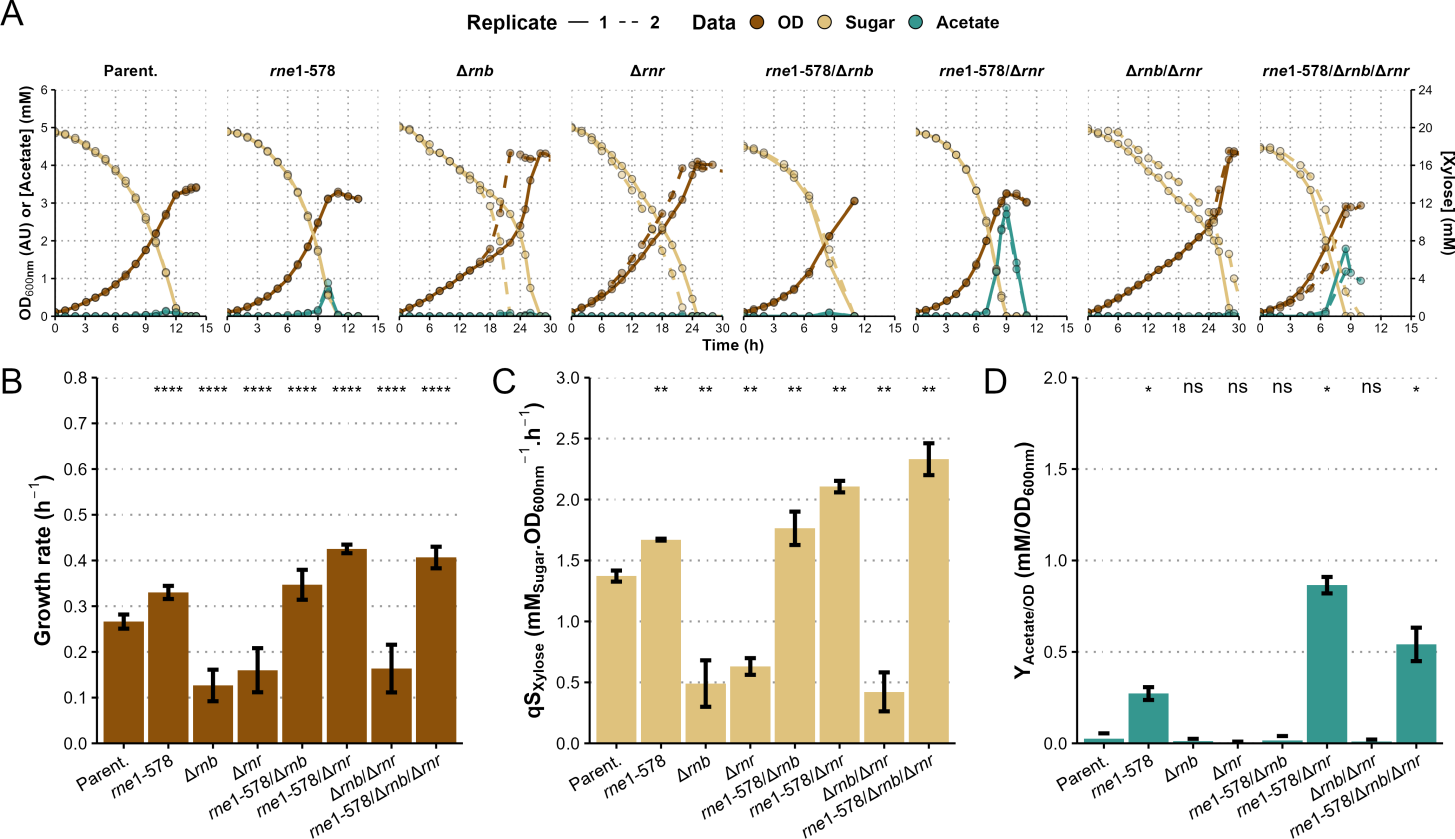
Kinetic analysis of *E. coli* RNase E, II, and R mutants grown on xylose in flasks. (**A**) Optical density (brown), xylose concentration (beige), and acetate concentration (green) as a function of time (*n* = 2 biological replicates). (**B**) Maximum growth rate (*n* ≥ 4 biological replicates) and (**C**) maximum specific xylose uptake rate (*n* = 2 biological replicates × 3 exponential-phase points) determined in the exponential growth phase. (**D**) Maximum acetate yield (*n* = 2 biological × 2 technical replicates) determined at the acetate peak. Bars represent mean ± SD.

The effects of RNase E, II, and R on growth and metabolism were investigated using the same protocol but on xylose, a non-preferred carbon source (Fig. 2).

Mutating RNase E, II, and R had very different effects on xylose than on glucose. Unexpectedly, all mutants with a truncated RNase E C-terminal domain showed accelerated growth. The growth rate changes associated with the RNase E truncation were highly dependent on the carbon source (Fig. S1). The maximum growth rates of *rne*1-578 and *rne*1-578/Δ*rnb* on xylose were approximately 26% higher than the parental strain (µ of 0.33 ± 0.01 h^−1^ and 0.35 ± 0.03 h^−1^, respectively, compared with 0.27 ± 0.02 h^−1^), while an additional 24% increase was observed when RNase R was also deleted (µ of 0.43 ± 0.01 h^−1^ in the *rne*1-578/Δ*rnr* double mutant and 0.41 ± 0.02 h^−1^ in the triple mutant). In contrast, single or double deletions of RNase II and R negatively affected growth, with growth rates reduced by half compared with the parental strain (Fig. 2B). Changes in the maximum xylose consumption rates, qS_xylose_, mirrored those in maximum growth rates (Fig. 2C); namely higher consumption in mutants with a truncated RNase E C-terminal domain, even higher in strains also missing RNase R, but much lower in strains missing RNase II and/or RNase R. A marked increase in acetate production (peak concentration and yield) was observed in the *rne*1-578/Δ*rnr* double mutant compared to single mutants (Fig. 2A and D). Altogether, these results show that truncation of the C-terminal domain of RNase E is sufficient to improve growth and sugar uptake on xylose, which may lead to increased acetate production. RNase R deletion, which in isolation has a deleterious effect on growth and xylose uptake, unexpectedly further accelerates growth and boosts xylose uptake and acetate production in conjunction with C-terminal RNase E truncation, demonstrating a strong synergistic interaction between the two mutations. These results also show that the effects of RNase E, II, and R on growth, sugar uptake, and acetate production vary depending on the carbon source.

### RNase E regulates the production of glycogen, a key compound in carbon storage, alone and in synergy with RNase R

Acetate production provides information on carbon balance in the lower part of glycolysis. In the upper part of glycolysis, *E. coli* can also produce glycogen from glucose-6-phosphate to store carbon.

**Fig 3:**
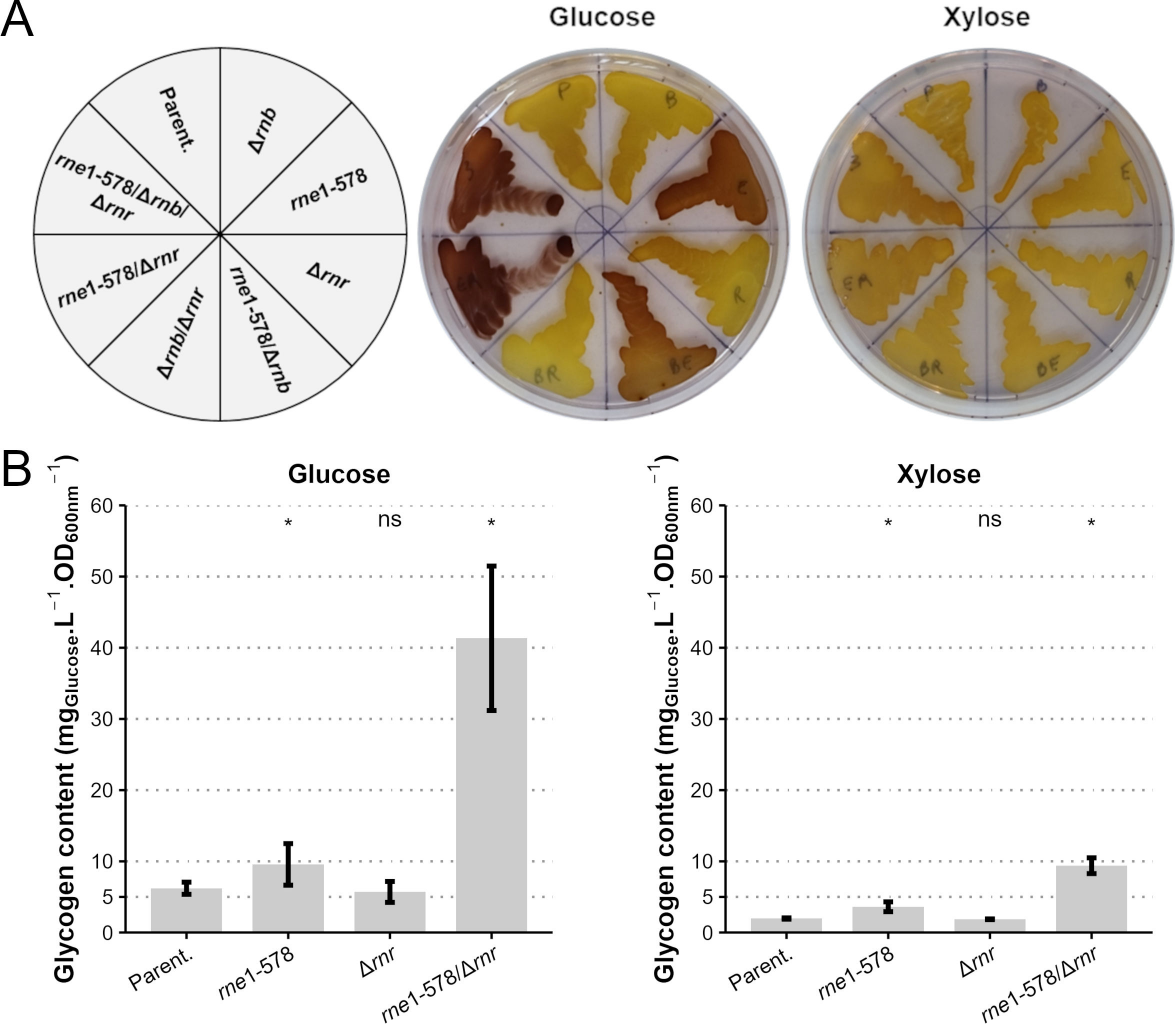
Quantification of glycogen content in *E. coli* RNase mutants grown on glucose or xylose. (**A**) (Left) Locations of the different mutants in the Petri dishes. (Middle) Qualitative measurement of glycogen content after 20 h growth on glucose and (right) 24 h growth on xylose. Glycogen content is reflected by color intensity: darker brown staining indicates greater glycogen accumulation (*n* = 2 biological replicates). (**B**) Enzymatic quantification of glycogen concentration during exponential growth on glucose (left) and xylose (right) in *rne*1-578, Δ*rnr*, and *rne*1-578/Δ*rnr* mutants. The selected strains represent low (yellow), medium (light brown), or high (dark brown) glycogen concentrations. Bars represent mean ± SD (*n* = 4 biological replicates).

On glucose, three levels of glycogen content were clearly visible after iodine staining in Petri dishes (Fig. 3A): (i) no/low content in the parental strain as well as in the Δ*rnb*, Δ*rnr,* and Δ*rnb*/Δ*rnr* mutants, (ii) moderate content in the *rne*1-578 and *rne*1-578/Δ*rnb* mutants, and (iii) high content in *rne*1-578/Δ*rnr* and the triple mutant. On xylose, however, no glycogen was detected with this method (Fig. 3A, right panel).

While glycogen accumulation was qualitatively assessed in all strains via iodine staining, quantitative enzymatic measurements were performed on selected representative mutants using glucose and xylose to validate the three phenotypic classes. Intracellular glycogen concentrations measured for each level of glycogen accumulation (in parental strain and Δ*rnr* for low glycogen content*, rne*1-578 for moderate glycogen content, and *rne*1-578/Δ*rnr* for high glycogen content) confirmed the observed stepwise increases, with up to 6.7-fold higher glycogen accumulation in the double mutant on glucose (Fig. 3B). Surprisingly, the same pattern was observed on xylose (Fig. 3B), although the glycogen concentrations were in each strain approximately four times lower than those for growth on glucose. These results show that C-terminal truncation of RNase E contributes to regulating glycogen accumulation from glucose and xylose, both in isolation and to an even greater extent in combination with RNase R deletion.

### Metabolic changes associated with RNase E, II, and R mutations are not correlated with variations in ATP levels

**Fig 4:**
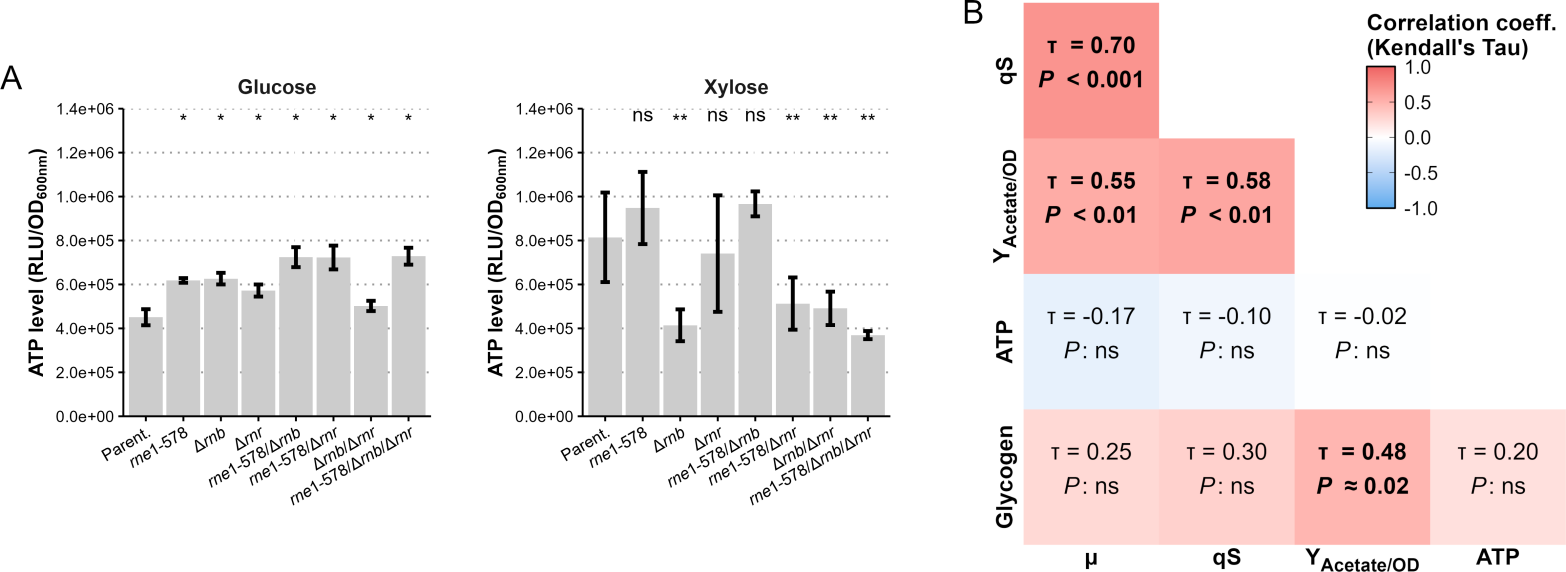
ATP levels in *E. coli* RNase mutants grown on glucose or xylose and their relationship with growth and metabolic parameters. (**A**) ATP levels measured during exponential growth on glucose (left) and xylose (right). Bars represent the mean ± SD of *n* ≥ 4 biological replicates. (**B**) Correlation matrix of growth and metabolic parameters in the parental strain and RNase mutants. Pairwise correlations were calculated between maximal growth rates (µ, in h^−1^), maximum specific substrate consumption rates (qS, in mM_carbon_.OD^−1^.h^−1^), acetate yields (Y_Acetate/OD_), intracellular glycogen contents (glycogen, qualitative level), and intracellular ATP levels (ATP, in RLU/OD). The values shown are Kendall’s correlation coefficient (τ) and the associated *P* value.

Having observed that RNases play an important role in regulating growth and metabolism, notably carbon storage metabolism, we wondered whether these changes might be correlated with cellular energy. We measured the intracellular ATP level in exponentially growing cells to approximate their cellular energy status (Fig. 4A). On glucose, intracellular ATP levels were slightly higher in the mutants compared with the parental strain. On xylose, four mutants (with single deletion of RNase II, double deletion of RNase II and R, the *rne*1-578/Δ*rnr* double mutant, and the triple mutant) had significantly reduced ATP levels compared with the parental strain. The correlation matrix of all growth and metabolic data collected during the study (growth and specific substrate consumption rates, acetate yields, glycogen contents, and ATP levels determined in the parental strain and the seven mutants on glucose or xylose) is shown in Fig. 4B (correlation coefficients and *P* values) and Fig. S2 (scatter plots).

As expected, moderate to strong significant correlations (τ > 0.50; *P* < 0.01) were observed between growth rates, specific substrate consumption rates, and acetate concentrations, but none of these three parameters or glycogen content were significantly correlated with ATP levels. These results suggest that the changes observed in the growth and metabolism of RNase mutants are not related to changes in cellular energy status.

### Combination of C-terminal truncation of RNase E and deletion of RNase II results in recombinant protein overproduction and maintained metabolic performance

**Fig 5:**
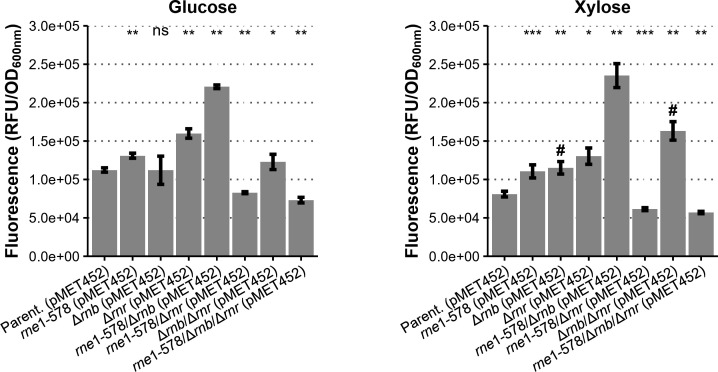
Production of msfGFP in *E. coli* RNase mutants grown on glucose or xylose. All strains were transformed with the pMET452 plasmid expressing the fluorescent protein msfGFP and grown on glucose or xylose for 22 h, except for the Δ*rnb* (pMET452) and Δ*rnr* (pMET452) single mutants and the Δ*rnb*/Δ*rnr* (pMET452) double mutants, which were grown for 32 h on xylose. The maximum values of msfGFP fluorescence/OD reached at the end of the culture on glucose (left) and on xylose (right) are plotted for all strains; the strains annotated “#” did not reach the stationary phase within 32 h. Bars represent the mean ± SD of *n* ≥ 3 biological replicates.

Given their broad effects on growth and metabolic regulation on two different carbon sources, we investigated whether these RNase mutations also affect cellular performance under biotechnologically relevant conditions in *E. coli*, such as under recombinant protein production. We used msfGFP as a reporter for recombinant protein production. This fluorescent protein offers the advantage of rapid folding, high stability, and easy *in situ* quantification. Fluorescence intensity was therefore used to evaluate protein production levels. We transformed the *E. coli* BL21(DE3) parental strain and all mutants with the pMET452 plasmid expressing the fluorescent protein msfGFP. Growth and fluorescence were monitored on microplates over time (Fig. S3). The fluorescence production maxima measured at the end of the cultures (22 h or 32 h for the slowest growth conditions) are plotted in Fig. 5.

msfGFP production differed significantly relative to the parental strain in all seven mutants, on glucose and on xylose, except in the single Δ*rnb* (pMET452) mutant on glucose. The same trends were observed on glucose (Fig. 5, left panel) and on xylose (Fig. 5, right panel): no change or a slight increase in msfGFP production for the three single mutants and the Δ*rnb*/Δ*rnr* (pMET452) double mutant, a 24%–34% decrease for the *rne*1-578/Δ*rnr* (pMET452) double mutant and the triple mutant, and an approximately twofold to threefold increase for the *rne*1-578/Δ*rnb* (pMET452) double mutant. The magnitude of the increase observed in the *rne*1-578/Δ*rnb* (pMET452) double mutant is remarkable given the relatively weak effects of the two mutations in isolation, and their combined effect demonstrates strong synergy between RNase E and II in regulating recombinant protein production. Given the high msfGFP production in the RNase E and RNase II double mutant, we examined the growth and metabolic dynamics of *rne*1-578/Δ*rnb* (pMET452) relative to BL21(DE3) (pMET452) in flask cultures (Fig. S4). The kinetic parameters, glycogen content and fluorescence productivity measured in the two strains are listed in Table 1.

**TABLE 1:** Kinetic parameters, glycogen content, and fluorescence production in *E. coli* BL21(DE3) (pMET452) and *rne*1-578/Δ*rnb* (pMET452) grown on glucose or xylose^*a*^

Sugar	Parameter	Parent (pMET452) (mean ± SD)	*rne*1-578/∆*rnb* (pMET452) (mean ± SD)	Ratio	*P^b^*
Glucose	µ (h^−1^)	0.65 ± 0.01	0.58 ± 0.01	0.90	***
	qS (mM_Glc_.OD_600 nm_^−1^.h^−1^)	2.38 ± 0.06	1.97 ± 0.05	0.83	***
	Y_Acetate/OD_ (mM/OD_600 nm_)	1.44 ± 0.12	0.88 ± 0.02	0.61	*
	Glycogen (mg_Glc_.L^−1^.OD_600 nm_^−1^)	6.80 ± 0.29	19.35 ± 0.27	2.84	*
	Fluorescence/OD_600 nm_ (AU)	27,929 ± 791	55,147 ± 762	1.97	*
Xylose	µ (h^−1^)	0.28 ± 0.01	0.34 ± 0.01	1.22	**
	qS (mM_Xyl_.OD_600 nm_^−1^.h^−1^)	1.51 ± 0.01	1.76 ± 0.06	1.16	*
	Y_Acetate/OD_ (mM/OD_600 nm_)	0.16 ± 0.21	0.04 ± 0.01	0.22	ns
	Glycogen (mg_Glc_.L^−1^.OD_600 nm_^−1^)	0.71 ± 0.63	3.02 ± 0.61	4.24	*
	Fluorescence/OD_600 nm_ (AU)	55,351 ± 762	87,568 ± 4,744	1.58	*

^*a*^ All measurements were performed in the exponential phase except for the maximum acetate yield (Y_Acetate/OD_), which was determined at the acetate peak. µ, maximum growth rate; qS, maximum substrate consumption rate. Results are the mean ± SD of three biological replicates.

^*b*^ ns*, P* > 0.05; *P ≤ 0.05; **P ≤ 0.01; ***P ≤ 0.001; ****P ≤ 0.0001.

The results in Table 1 confirm the increased productivity of the msfGFP in the double mutant compared with the parental strain. The major metabolic traits triggered by the double mutations of RNase E and RNase II in *rne*1-578/Δ*rnb*, namely accelerated growth on xylose (Fig. 2B) and increased glycogen accumulation on glucose (Fig. 3), were also observed in *rne*1-578/Δ*rnb* (pMET452) when overexpressing msfGFP (Table 1).

**Fig 6:**
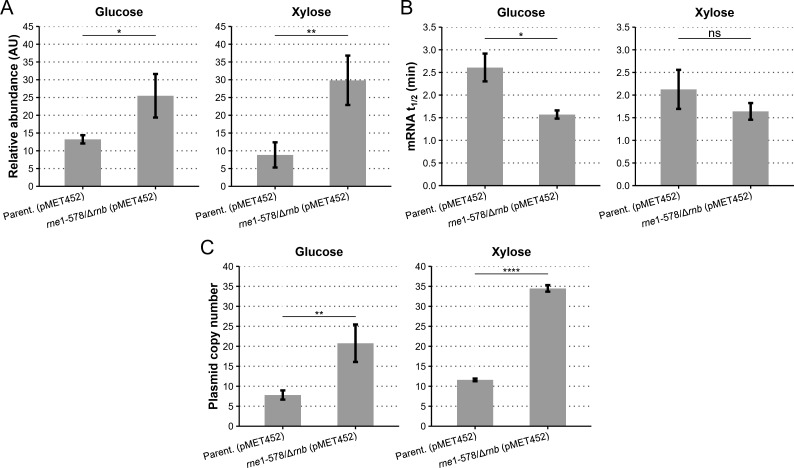
Quantification of *msfGFP* mRNA abundance and stability, and plasmid copy number in *rne*1-578/Δ*rnb* (pMET452) and BL21(DE3) (pMET452) during exponential growth on glucose or xylose. (**A**) Relative abundance of *msfGFP* mRNA. (*n* = 2 biological × 2 technical replicates). (**B**) Half-life (t_1/2_) of *msfGFP* mRNA (*n* = 2 biological replicates, including one tested with *n* = 2 technical replicates). (**C**) pMET452 copy number (*n* = 2 biological × 2 technical replicates). Bars represent mean ± SD.

We investigated the mechanism underlying msfGFP overproduction in the *rne*1-578/Δ*rnb* (pMET452) mutant by measuring *msfGFP* mRNA abundance and half-life during the exponential growth on glucose or xylose. The variation in msfGFP fluorescence in the *rne*1-578/Δ*rnb* (pMET452) mutant was similar to that of *msfGFP* mRNA levels on both substrates: a 1.5- or 2-fold increase in fluorescence (Table 1) was associated with a 2- or 3-fold increase in *msfGFP* mRNA levels (Fig. 6A) compared with the parental strain. However, this increase in *msfGFP* mRNA levels was not due to increased transcript stability: the *msfGFP* mRNA half-lives were 2.6 ± 0.3 min and 1.6 ± 0.1 min for the parental strain and the mutant, respectively, on glucose, and 2.1 ± 0.4 min and 1.6 ± 0.2 min on xylose (Fig. 6B). We then compared the plasmid copy number (PCN) in *rne*1-578/Δ*rnb* (pMET452) and the parental strain BL21(DE3) (pMET452) (Fig. 6C). The number of plasmid copies was 2.7-fold higher on glucose and 3-fold higher on xylose in the RNase E/RNase II double mutant compared to the parental strain, indicating that the elevated levels of msfGFP mRNA and protein in the double mutant were directly due to the increased plasmid copy number.

## DISCUSSION

This study elucidates the individual contributions of RNase E, II, and R to the regulation of *E. coli* growth and metabolism and reveals unexpectedly strong synergistic effects among the three enzymes.

Individually, the studied RNase mutations did not strongly affect the growth or sugar uptake on glucose. On xylose, however, C-terminal truncation of RNase E led to increased growth and sugar uptake, whereas deletion of RNase II or RNase R had the opposite effects. This suggests that alternative RNases compensate for the loss of RNase II or R on glucose but not on xylose. Remarkably, however, the highest growth rates on xylose were observed when RNase E truncation was combined with RNase R or RNase II deletion, evidencing a strong synergistic effect. The faster growth of the RNase E/RNase II double mutant was also observed under conditions of high recombinant protein production, and the acceleration/deceleration of growth in mutants did not appear to result from changes in intracellular ATP levels, a proxy for cellular energy state. In contrast, perturbation of the RNA machinery by deletion of the poly(A) polymerase PAP I has been shown to lead to decreases in both ATP levels and growth rates on glucose (2). It would be interesting to identify which RNAs (mRNAs and/or regulatory small RNAs) are differentially stabilized or degraded in the single and combined mutants to clarify the molecular basis of the growth rate variations observed on xylose.

Our results for acetate production further emphasize the individual and joint effects of the RNases. While isolated mutations in RNase II and RNase E increased acetate production on glucose and xylose, respectively, the RNase E and RNase R mutations in combination had the synergistic effect of enhancing acetate production on both substrates. Acetate overflow is a complex mechanism potentially induced by unbalanced flux between glycolysis and the TCA cycle. In the *E. coli* B parental strain BL21(DE3), low acetate production is related to an efficient TCA cycle, due particularly to an active glyoxylate shunt (19, 20). In the RNase E mutant, the increased acetate production may result from a reduced flux in the phosphoenolpyruvate carboxylase connecting glycolysis to the TCA cycle, as previously reported in *E. coli* K12 upon RNase E perturbation (21). This interpretation is further supported by the overproduction of pyruvate, a metabolite like acetate that indicates a flux imbalance between glycolysis and the TCA cycle, which is observed following deletion of RNase G, a homolog of RNase E (22). Additionally, acetate production does not solely reflect increased synthesis flux but also depends on the balance between acetate excretion and reutilization. In *E. coli* K12, acetate can be reassimilated even under glucose excess through the reversible Pta-AckA pathway (23), suggesting that alterations in this pathway may also contribute to the increased acetate production observed in our mutants. RNase E, II, and R are therefore all implicated in the regulation of central carbon metabolism, most likely acting at the acetyl-CoA node.

C-terminal truncation of RNase E alone, but even more so in combination with deletions of RNase R and/or RNase II, was found to regulate carbon storage by substantially increasing glycogen accumulation, even in cells producing high levels of recombinant protein. The stronger effect on glucose than xylose may reflect higher levels of the glycolytic intermediate fructose-1,6-bisphosphate, an allosteric activator of ADP-glucose pyrophosphorylase, the enzyme that catalyzes the rate-limiting step of glycogen biosynthesis (24). The link between RNase E activity and glycogen production has previously been described in *E. coli* K12 and found to involve the carbon storage system (CSR): *glgCAP* mRNA, which encodes the glycogen synthesis enzymes, is a target of translational repression by CsrA (25), and RNase E indirectly regulates CsrA activity by cleaving its antagonist small RNA CsrB (26). Glycogen accumulation in our C-terminal truncated RNase E mutant may therefore be driven by a CSR-dependent glycogen production mechanism. However, how RNase R could contribute to this mechanism is unclear. Further studies are required to investigate the underlying molecular mechanism driving the synergistic effects of RNase E and R on the regulation of glycogen biosynthesis.

We found that C-terminal truncation of RNase E moderately enhanced recombinant protein production on both substrates, consistent with the common use of *E. coli* BL21 Star (DE3) (Invitrogen) to express truncated *rne*131 RNase E for heterologous protein production. However, a particularly striking result was the synergistic effect of RNase E truncation and RNase II deletion on recombinant protein production, with 1.7-fold and 2.1-fold higher protein production than the single RNase E mutant on glucose and xylose, respectively. Increased msfGFP production in the double mutant was not associated with enhanced *msfGFP* mRNA stability. Previous studies have also shown that C-terminal truncation of RNase E does not systematically lead to stabilization of the mRNA of interest (27). We showed that the higher levels of *msfGFP* mRNA and protein in the RNase E/RNase II double mutant were due to an increased number of plasmid copies. The replication of ColE1-derived origins, such as p15A, which was used in this study, is controlled by two small RNAs, RNA I and RNA II. RNA I stability is known to depend on both PAP I-mediated polyadenylation and RNase E cleavage (28). The manner in which RNase II may act synergistically with RNase E to regulate the stability of RNA I, and possibly of RNA II, remains to be elucidated. RNase II has been shown to associate with degradosome components (29), but to our knowledge, no information is available on the combined activities of RNase E and II on RNA I and II.

In conclusion, the strong synergistic effects of combined RNase mutations on *E. coli* growth and metabolism evidenced here open new avenues for developing *E. coli* BL21(DE3) chassis for biotechnological applications. The RNase E/RNase R double mutant is particularly attractive: (i) its rapid growth on xylose is highly unusual among BL21(DE3)-derived strains and can be used to enhance the bioprocess productivity using biomass-derived carbon sources such as xylose; (ii) its increased acetate production is advantageous when acetate is the desired product, for example, to serve as carbon feedstock for industrial biotechnology (30); and (iii) its high glycogen content indicates strong adaptability to stress conditions (31). Meanwhile, the RNase E/RNase II double mutant produced very high levels of recombinant protein without impaired cell growth or glycogen accumulation. This ability to tolerate the metabolic burden associated with high-level recombinant protein expression is promising for the efficient overproduction of heterologous proteins. We note finally that the triple mutant with all three RNase E, RNase R, and RNase II mutations did not outperform the RNase E/RNase R or RNase E/RNase II double mutants.

## MATERIALS AND METHODS

### Strains and plasmids

The *rne* truncation was introduced into *E. coli* strain BL21(DE3), by amplifying the *rne*1-598–FLAG–cat cassette from strain TM529 using the *rne*F/*rne*R primer pair (Table S1), and integrated by λ red recombination. Insertion of a FLAG tag within the disordered C-terminal region of RNase E is commonly used in the literature and does not appear to significantly affect its activity (32, 33); therefore, its presence in our construct is unlikely to be a major factor contributing to the phenotypes observed in our study. Chloramphenicol-resistant transformants were selected, and the resulting cassette was subsequently transferred into the parental BL21(DE3) strain by P1 transduction. Δ*rnb* or Δ*rnr* mutations were carried out in the parental BL21(DE3) strain using λ red recombination (34) and the primer pairs ∆*rnb*_SP1/∆*rnb*_SP2 and ∆*rnr*_SP1/∆*rnr*_SP2, respectively (Table S1). These deletions, verified by sequencing, removed the coding sequence from the second to the penultimate amino acid of the *rnb* and *rnr* genes. The double mutants, BL21(DE3) *rne*1-578/Δ*rnb,* BL21(DE3) *rne*1-578*/*Δ*rnr*, and BL21(DE3) Δ*rnb/*Δ*rnr*, and the corresponding triple mutant, were created by P1 transducing the Δ*rnb* or Δ*rnr* mutations to recipient BL21(DE3) *rne*1-578, BL21(DE3) Δ*rnb,* or BL21(DE3) *rne*1-578/Δ*rnr* strains from donor strains BL21(DE3) Δ*rnr* or BL21(DE3) Δ*rnb*. The antibiotic-resistance cassette was removed by FLP recombination. Mutations were confirmed by PCR using the primers listed in Table S1 and by sequencing. Sequencing of the genomic *rne* locus in the truncated RNase E mutants revealed a shift in the truncation, with an additional 20 C-terminal amino acids removed during construction, resulting in a *rne*1-578 mutant rather than *rne*1-598 (Fig. S5). Sixteen nucleotide substitutions were detected in the coding sequence, none leading to any change in the amino acid sequence. For recombinant protein production, the parental strain and its mutants were transformed with pMET452, which expresses the fluorescent protein msfGFP. pMET452 is a derivative of P_BAD_-*msfGFP* (35). The plasmid carries a p15A origin of replication (obtained from the plasmid pBMK11) and an *msfGFP* gene under the control of the constitutive P*_ompA_* promoter of *E. coli* MG1655. pMET452 was verified by whole-plasmid sequencing. All strains and plasmids used in the study are listed in Table 2, and primers are listed in Table S1.

**TABLE 2:** Strains and plasmids used in this study

Strain or plasmid	Genotype or features	Reference or source
Strains		
BL21(DE3)	*E. coli* B, *lon-11, Δ(ompT-nfrA)885, Δ(galMybhJ)884, λDE3 [lacI, lacUV5-T7 gene 1, ind1, sam7, nin5], Δ46, [mal+] K-12(λS), hsdS10*	(36)
BL21(DE3) Δ*rnb*	BL21(DE3), Δ*rnb*	This work
BL21(DE3) *rne*1-578	BL21(DE3), *rne*1-578-FLAG	This work
BL21(DE3) Δ*rnr*	BL21(DE3), Δ*rnr*	This work
BL21(DE3) *rne*1-578/Δ*rnb*	BL21(DE3), *rne*1-578-FLAG*,* Δ*rnb*	This work
BL21(DE3) Δ*rnb*/Δ*rnr*	BL21(DE3), Δ*rnb*, Δ*rnr*	This work
BL21(DE3) *rne*1-578*/*Δ*rnr*	BL21(DE3), *rne*1-578-FLAG, Δ*rnr*	This work
BL21(DE3) *rne*1-578/Δ*rnb/*Δ*rnr*	BL21(DE3), *rne*1-578-FLAG, Δ*rnb,* Δ*rnr*	This work
TM529	W3110 *mlc, rne*1-598*-*FLAG*-cat*	(37)
Plasmids		
P_BAD_-*msfGFP* plasmid	pBR322 origin, *araBAD* promoter, ampicillin resistance, *msfGFP*	(35)
pBMK11	pACYC184 derivative with p15A origin	(38)
pMET452	p15A origin, *ompA* promoter, ampicillin resistance, *msfGFP*	This work

### Growth conditions

The strains were grown in M9 minimal medium, as previously described (39). M9 medium was supplemented with 16.7 mM glucose or 20 mM xylose (100 mM carbon equivalent). The medium was supplemented with 100 µg/mL ampicillin if required. Erlenmeyer flasks were inoculated at an initial OD of 0.1. The temperature was set to 37°C and stirring at 150 rpm. OD measurements were performed with a Jenway 7200 spectrophotometer (Cole-Parmer). The strains were cultured in 96-well microplates in a Synergy H1 microplate reader (BioTek) at 37°C, in either 100 µL or 200 µL of M9 glucose (stirring at 425 cpm) or xylose (stirring at 807 cpm), with an initial OD ≈ 0.05. Volume changes and shaking were used to prevent cell sedimentation. Microplate OD measurements were normalized relative to the blank values for each medium and adjusted to a common OD scale.

### Fluorescence measurements

For cultures in microplates, msfGFP fluorescence (excitation 475 nm and emission 512 nm) was monitored every 25 min in the cultures using a Synergy H1 microplate reader (BioTek). The fluorescence values were normalized to the blank for each medium and adjusted to allow comparisons between measurements from cultures grown in 200 and 100 µL of medium. Fluorescence was also measured at the end of growth (approximately 22 h on glucose and 32 h on xylose) and normalized by the OD. For the cultures in Erlenmeyer flasks, fluorescence was measured during exponential growth at an OD of approximately 1 with the Synergy H1 microplate reader (BioTek).

### Analytical methods

Glucose, xylose, and acetate concentrations were quantified by HPLC using refractive index and ultraviolet detection. The device was equipped with a Bio-Rad HPX87H column maintained at a temperature of 48°C. The eluent was 5 mM H_2_SO_4_ at a flow rate of 0.5 mL/min.

### Glycogen assays

Glycogen contents were determined using iodine vapor staining (40). After overnight liquid culture in M9 glucose or xylose, the cells were washed with 9 g/L NaCl, resuspended in M9 glucose or xylose to an OD of 1 AU, and 10 µL were spread (8 cm^2^) on Petri dishes. The cells were left to dry before being incubated overnight. The Petri dishes were placed in a sealed chamber containing iodine crystals for approximately 5 min to reveal the presence of glycogen. Enzymatic assays of intracellular glycogen concentrations were performed as previously described (41). Briefly, cells were harvested in the exponential phase (OD of between 0.7 and 1), washed, pelleted, and lysed. The glycogen from this lysate was hydrolyzed using amyloglucosidase, and the glucose subunits were quantified using glucose assay kit GAGO20 (Sigma-Aldrich). Absorbance at 490 nm was measured using a Synergy H1 microplate reader (BioTek).

### ATP assay

Intracellular ATP levels were determined using the BacTiter-Glo Microbial Cell Viability Assay Kit (Promega) as described previously (42). Briefly, 0.8 mL of culture was collected and centrifuged, and the cell pellet was resuspended in 0.8 mL of fresh medium. Samples (75 µL) were transferred into a black 96-well microplate filled with 125 µL of BacTiter Glo reagent. Luminescence was measured after 5 min of incubation at room temperature with a Synergy H1 microplate reader (BioTek).

### RNA sampling and extraction

Cells collected at an OD of approximately 0.7 and immediately frozen in liquid nitrogen were used to determine mRNA abundances and to define the *T*_0_ level for half-life measurements. Rifampicin (0.5 g/L) was added immediately afterward to block transcription initiation, and cells were harvested at 0.5, 1.5, 2.5, 4, and 7 min, immediately frozen in liquid nitrogen, and stored at −80°C. The cells were thawed and mechanically lysed using glass beads. Total RNA was extracted using the RNeasy Mini Kit (Qiagen). DNA contamination was removed using DNase I treatment. Total RNA yields were similar across all samples, both on glucose (17.0 ± 1.2 µg RNA/mg of dry cell weight) and on xylose (11.0 ± 1.0 µg RNA/mg of dry cell weight). All samples had an RNA Integrity Number ≥7.5, as determined using Bioanalyzer 2100 (Agilent). rRNA content and integrity were comparable between the mutant and parental strains.

### Determination of mRNA abundance and half-life by RT-qPCR

At least 2.5 µg of total RNA was reverse transcribed using 1.5 U of SuperScript II reverse transcriptase with random primers (Invitrogen) and RNase H (Invitrogen). The resulting cDNA was then purified on Microspin G-25 columns (Cytiva). Quantitative PCR was performed with the primers indicated in Table S1, using IQTM SYBR Green Supermix (Biorad). Amplification-related fluorescence was measured with a LightCycler 480 II instrument (Roche). The specificity of the primers was verified on a standard range of *E. coli* BL21(DE3) genomic DNA or pMET452 plasmid DNA, with efficiencies between 97% and 111%. For each RNA species, strain, and condition, the linear regression coefficient (*k*′) of the quantification cycle (Cq) as a function of time and its associated coefficient of determination (*R*²) were calculated. *k*′ values were only retained if *R*² was greater than 0.8 (Fig. S6). The half-lives of the RNAs were then determined from *k*′ using the formula *t*_1/2_ = 1/*k*′. Abundances were calculated using the 2^−∆Cq^ formula relative to *ihfB* mRNA (43). Additional parameters not reported in the main text are listed in Table S2 according to the minimum information for publication of quantitative RT-PCR experiments (MIQE) guidelines.

### PCN determination

Cells were harvested at OD ~0.7 and immediately frozen in liquid nitrogen. Samples were normalized to an OD of 1 AU, and 200 µL was pelleted and resuspended in 200 µL of solution 1 (50 mM Tris, pH 7.5, 50 mM EDTA, 20% sucrose, 1 mg/mL RNase A, and 50 µg/mL proteinase K), and incubated at room temperature for 10 min. Then, 200 µL of solution 2 (50 mM Tris, pH 7.5, 50 mM EDTA, 20% sucrose, and 5% SDS) was added. Finally, the samples were incubated for 10 min at 70°C before dilution for qPCR. Two genes were targeted: a chromosomal gene (*maeA*, located relatively close to the replication termination site) and a plasmid gene (*msfGFP*). PCN was calculated as PCN = (*E_maeA_*^Cq*maeA*^)/(*E_msfGFP_*^Cq*msfGFP*^), where *E* and Cq correspond to amplification efficiency and quantification cycle values for each gene (44). Additional parameters are listed in Table S2 according to MIQE guidelines.

### Data processing and statistical analysis

Growth rates were determined by measuring the slope of the linear portion of ln(OD) versus time curves (i.e., OD generally between 0.1 and 2). To calculate specific carbon source consumption rates (qS), OD and sugar concentration values were smoothed by polynomial regression. For qS calculation, three instantaneous qS values (qSinst, defined as the time derivative of the sugar concentration [dS/dt] normalized by the corresponding OD) were extracted from the exponential phase of each of the two biological replicates. All the six values (two replicates ×3 qSinst) were then used to calculate the mean and perform statistical comparisons between strains. The acetate yield per biomass (*Y*_Acetate/OD_, expressed in mM/OD) was calculated as the ratio of net produced acetate (maximum acetate concentration minus the initial concentration at *T*_0_) to the net increase in optical density (OD at peak acetate minus OD at *T*_0_), before its reconsumption.

A correlation matrix was built by calculating Kendall rank correlation coefficients between the averages of the physiological parameters of each strain for each carbon source. Kendall’s tau was chosen as the correlation metric because of its applicability to both ordinal and continuous variables. The correlation matrix and associated *P*-values were calculated in R using the stats 4.5.1 and rstatix 0.7.2 packages with default settings.

Results from the parental strain and all RNase mutants (Fig. 1B through D, Fig. 2B through D, Fig. 4A, and Fig. 5) were compared using Wilcoxon-Mann–Whitney tests with Benjamini–Hochberg correction. For clarity, only comparisons with the parental strain are shown in the graphs. Results from the BL21(DE3) (pMET452) strain and the *rne*1-578/Δ*rnb* (pMET452) mutant (Table 1; Fig. 6) were compared using Welch tests, after verifying the normality of the residuals for each parameter using Shapiro–Wilk tests and Q-Q plots. One-sided Wilcoxon–Mann–Whitney tests (*H*_1_: P[Mutant >Parent.]>0.5), consistent with our *a priori* directional hypothesis that mutants exhibit increased glycogen concentration and fluorescence production in mutants, with Hommel correction, were used to test increases in glycogen concentration and fluorescence production (Fig. 3B; Table 1). The number of data points (biological and/or technical replicates) used in each statistical test is indicated below the corresponding figures. Statistical significance was defined as *P* < 0.05, and *P* values were represented as follows on the figures: ns, *P* > 0.05; *P ≤ 0.05; **P ≤ 0.01; ***P ≤ 0.001; ****P ≤ 0.0001. All statistical tests were performed in R using the rstatix 0.7.2 package.

## Data Availability

All data generated or analyzed during this study are included in this published article and its supplemental material.

## SUPPLEMENTAL MATERIAL

Supplemental material aem.00090-26-s0001.docx Table S1; Fig. S1 to S6.

Table S2 aem.00090-26-s0002.xlsx Additional information on quantitative RT-PCR and qPCR experiments.

ASM does not own the copyrights to Supplemental Material that may be linked to, or accessed through, an article. The authors have granted ASM a non-exclusive, world-wide license to publish the Supplemental Material files. Please contact the corresponding author directly for reuse.
