# Supplementary material for: Synergistic and individual effects of RNase E, II, and R in the regulation of Escherichia coli growth and metabolism
Source: Appl Environ Microbiol. 2026 Jun 26;92(7):e00090-26. doi: 10.1128/aem.00090-26 (PMC13390384; doi:10.1128/aem.00090-26)
Supplement: Supplemental material — Table S1; Fig. S1 to S6. [file aem.00090-26-s0001.docx]

**Supplementary data**

**Table S1:** List of primers used in this study.

| **Name** | **Sequence (5’-3’)** | **Use** |
| --- | --- | --- |
| *msfGFP* F | CGATGGCCCTGTCCTTTTACCA | qPCR |
| *msfGFP* R | GCCATGTGTAATCCCAGCAGCA |  |
| *ihfB* F | GCCAAGACGGTTGAAGATGC |  |
| *ihfB* R | CAAAGAGAAACTGCCGAAACC |  |
| *maeA F* | GCTGGCCCTGTACTGCTGGAAT |  |
| *maeA R* | CCATGCTCGTTCCGCTTGTTC |  |
| p15A F | GCGCTAGCGGAGTGTATACTGGCT | Amplification of p15A ori from pBMK11 |
| p15A R | GGTAACGAATCAGACAATTGACGGCTTG |  |
| P*ompA* F | CTCACGCCACGAGACAACTTTCCGGG | Amplification of P*_ompA_* from *E. coli* MG1655 genome |
| P*ompA* R | ATGTAAAGTCTACAACGTAGTTGAAAACTTACAAGTG |  |
| *rnb* verif. F | GCCCATTCCCGTTTATTGAT | ∆*rnb* verification |
| *rnb* verif. *R* | CGCAGATTATTAATCTGCTGG |  |
| *rnr* verif. F | CTTTCTAAGGCCGTGCAA | ∆*rnr* verification |
| *rnr* verif. R | TGCGTGGATGCCGTAAAT |  |
| *rne*F | TGCCGTCTGAAGAAGAGTTCG | *rne* truncation for λ red recombination and verification |
| *rne*R | ATGCAGGGATTGTCGCTCTTC |  |
| ∆*rnb*_SP1 | acatattaaccttgccgcgtcagacagattcgcgtaaaactgtcagccgctctaatggccaccaaaatagacaattatg | ∆*rnb* for λ red recombination |
| ∆*rnb_*SP2 | aaaactgccgggatatgatgaaggtagagcggggaaataaacggcccattcataaggaatgggccgtgaaaggagatta |  |
| ∆*rnr_*SP1 | agaatcaaccgctttataaattattgctggtggagtgacgaaaatcttcatcagagatgacaacggaggaaccgagatg | ∆*rnr* for λ red recombination |
| ∆rnr_SP2 | cgttcacgccgcatccggcatgaacaaagcgcattttgtcagcaatctaaccctcttcttttaaagagggtattgatca |  |

**Table S2**: Additional information on quantitative RT-qPCR and qPCR experiments (excel file).

**
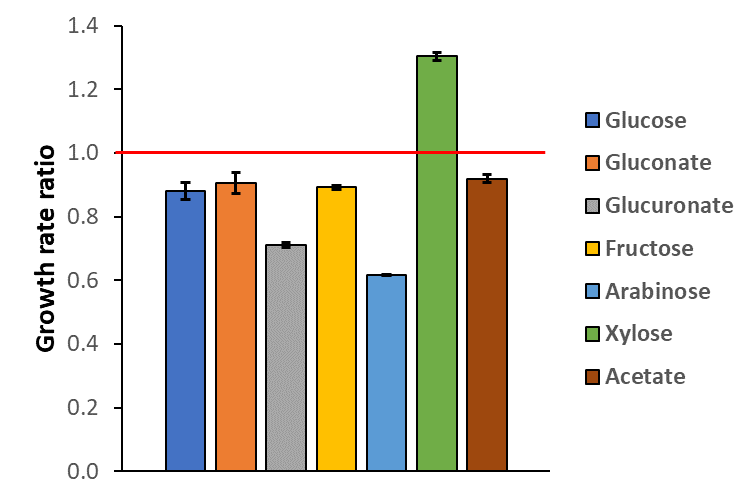
**

**Figure S1:** Growth rate ratio of the *rne*1‑578 RNase E mutant relative to the parental strain, measured in microplates in M9 synthetic medium supplemented with various carbon sources. All media contain 100 mM equivalent carbon concentration. The red line indicates a ratio of 1 relative to the parental strain; values above this line correspond to an increased growth rate in the truncated RNase E mutant. Bars represent the mean ± SD of n = 2 biological replicates.


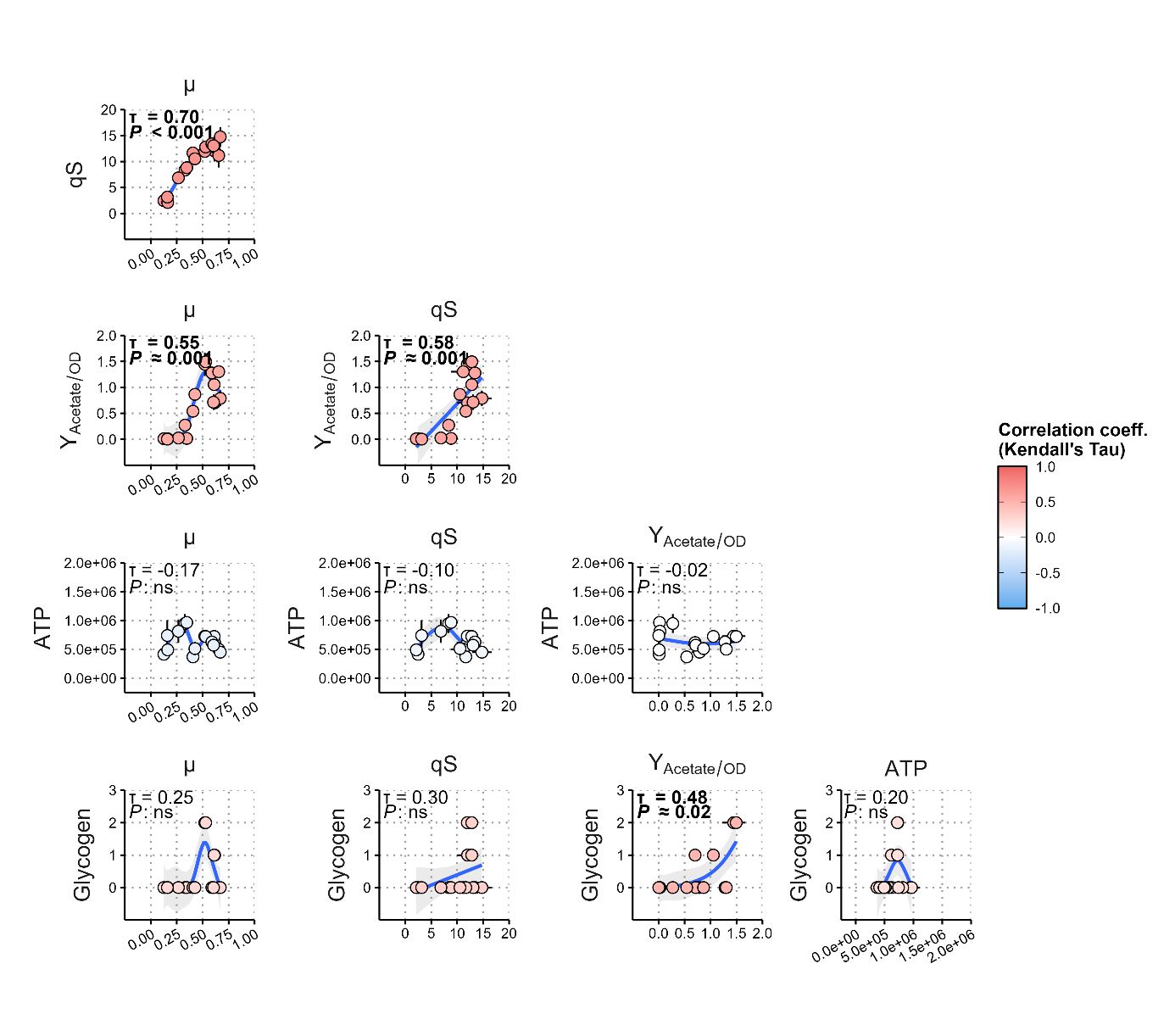


**Figure S2: Correlations between physiological parameters.** Calculation of Kendall's correlation coefficient (τ) between four quantitative parameters (growth rate, µ, substrate consumption rate, qS, acetate yield, Y_acetate/OD_, and ATP concentration in the exponential growth phase, ATP) and one qualitative parameter (glycogen level, Glycogen) (n = 16). The associated *P* values are also indicated on the graphs. Dots represent mean ± SD of each strain, either on glucose or on xylose (for quantitative variables, n ≥ 3). The blue curves are GAM regression lines (ggplot2 4.0.0 package; default parameters) illustrating the relationship between each pair of parameters.


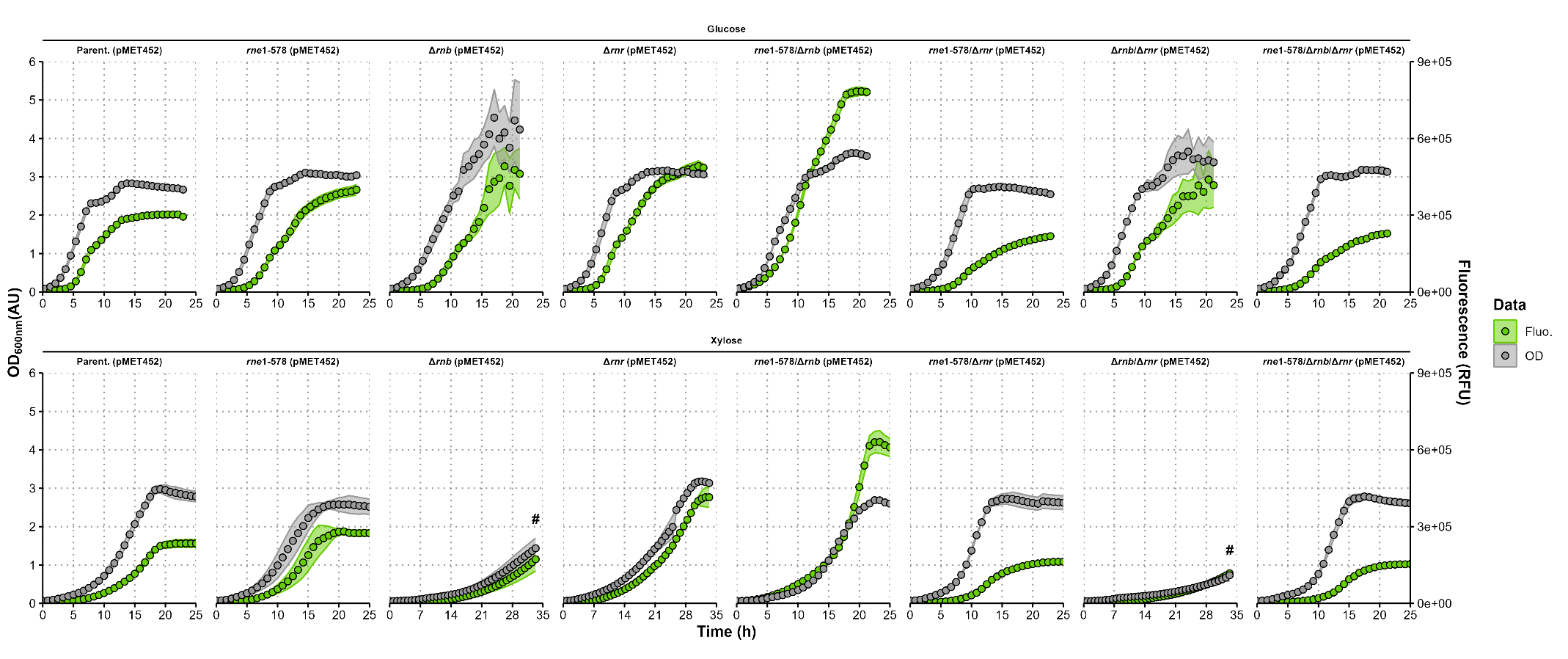


**Figure S3: Kinetic analysis of *E. coli* RNase E, II and R mutants grown on glucose or xylose** **on microplates**. All strains were transformed with the pMET452 plasmid expressing the fluorescent protein msfGFP. Optical density (grey) and fluorescence (green) versus time during growth on glucose (top) or xylose (bottom). The strains annotated “#” did not reach the stationary phase within 35 h. Dots and ribbons represent mean ± SD (n ≥ 3 biological replicates).


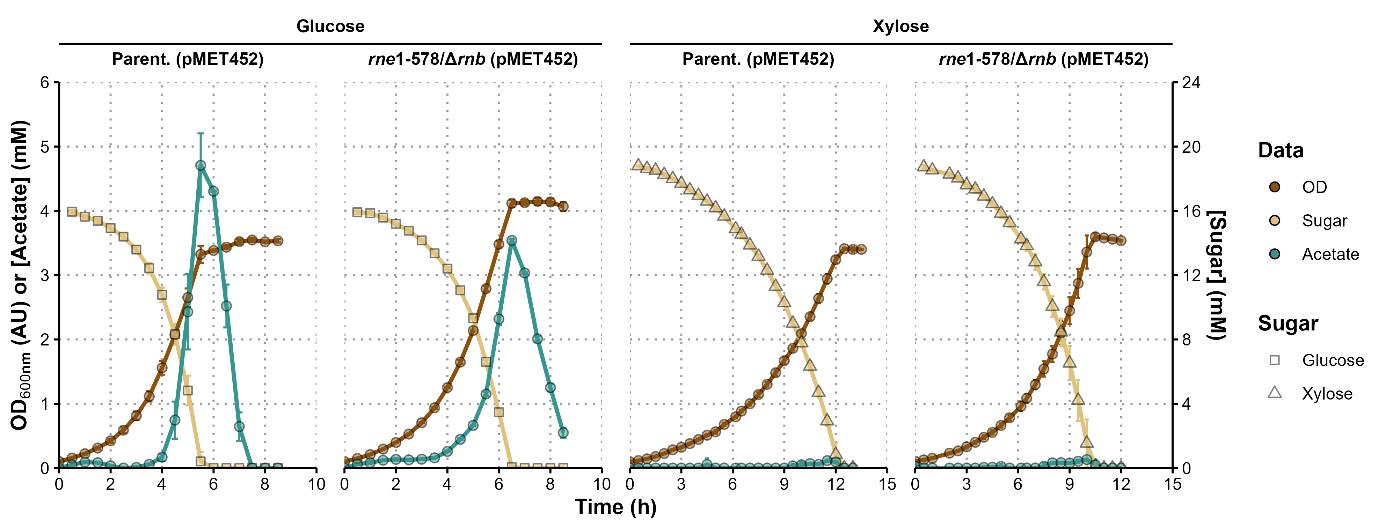


**Figure S4: Kinetic analysis of *E. coli* BL21(DE3) (pMET452) and *rne*1-578/Δ*rnb* (pMET452) grown on glucose or xylose in flasks.** Optical density (grey) glucose concentration (beige) and acetate concentration (green) versus time during growth on glucose (left) or xylose (right). Bars represent mean ± SD (n = 3 biological replicates).

**
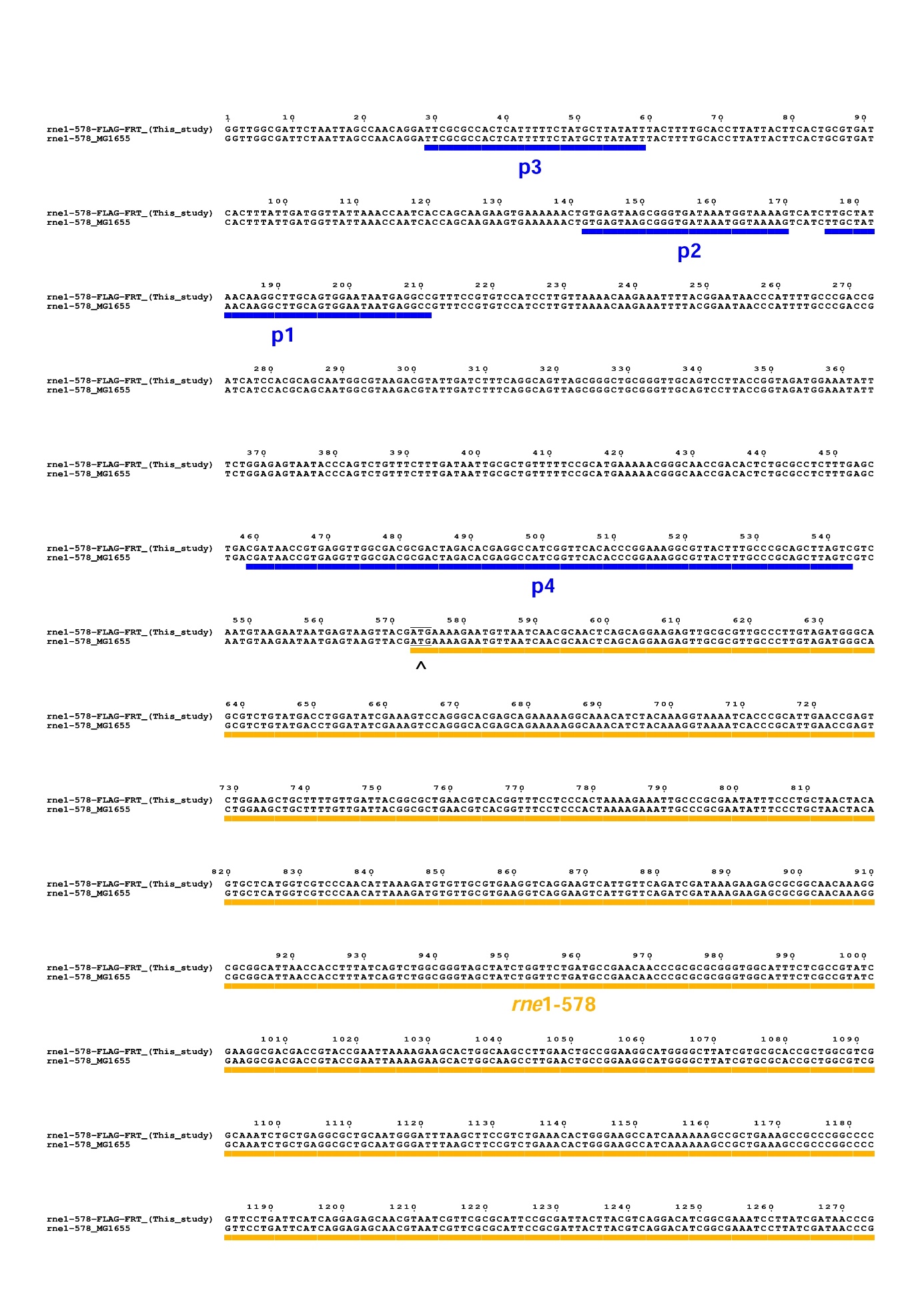

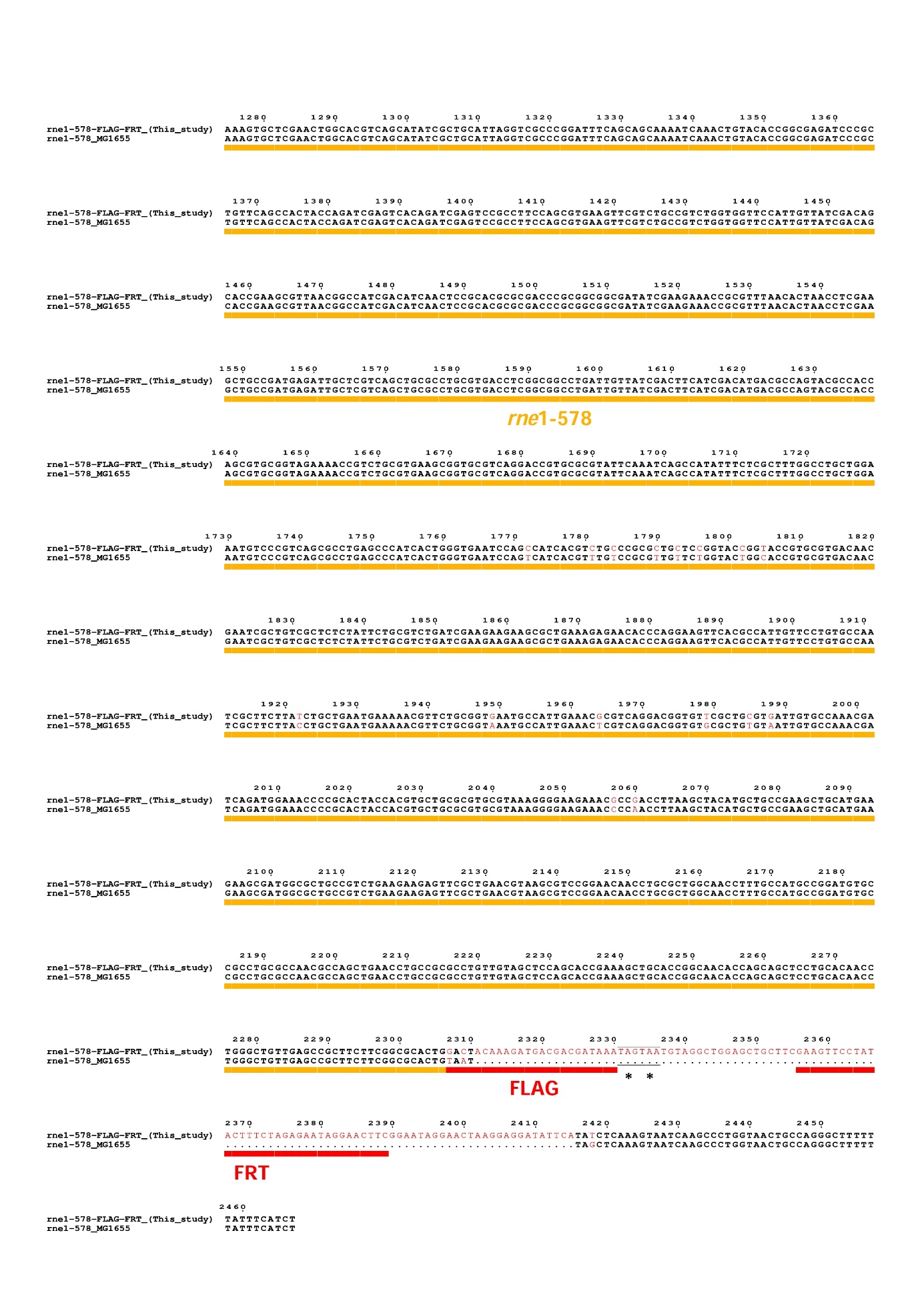
**

**Figure S5: Genomic *rne* locus.** The *rne* gene allele was sequenced using Illumina technology, and the C-terminal region was independently validated by Sanger sequencing. The promoters (-10 and -35), numbered p1 to p4 (Ecocyc data), are shown in blue. The *rne*1-578 coding sequence is underlined in orange, its start codon is marked with an arrow and the stop codons with stars. The FLAG sequence (positions 1735–1758) and the 34-bp minimal FRT sequence, 19 nucleotides downstream of the *rne* coding sequence, are highlighted in red. The sequence was aligned to the *rne* locus from strain MG1655 (GCF_000005845.2). Sixteen nucleotide substitutions were detected in the coding sequence, none leading to any change in the amino acid sequence.

**
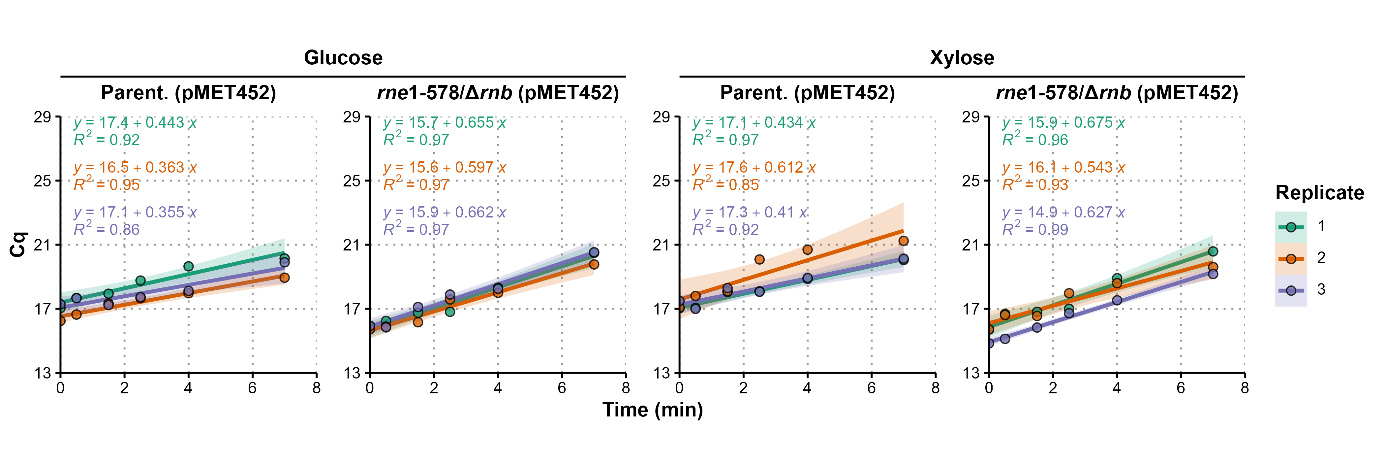
Figure S6: Quantification of *msfGFP* mRNA stability in *E. coli* BL21(DE3) (pMET452) and *rne*1-578/Δ*rnb* (pMET452) during exponential growth on glucose or xylose.** Calculation of *msfGFP* mRNA half-lives using linear regression on Cq values obtained from RNA samples collected between 0 and 7 min after addition of rifampicin. The half-life of *msfGFP* mRNA was calculated for strains BL21(DE3) (pMET452) and *rne*1-578/Δ*rnb* (pMET452) grown on glucose (left) and xylose (right). The linear regression equations and R² values are shown on the graphs (n = 2 biological replicates, including one tested with n = 2 technical replicates per strain and condition).
